# Bacterial endotoxin lipopolysaccharides regulate gene expression in human colon cancer cells

**DOI:** 10.1186/s13104-023-06506-9

**Published:** 2023-09-13

**Authors:** Heping Cao

**Affiliations:** United States Department of Agriculture, Agricultural Research Service, Southern Regional Research Center, 1100 Allen Toussaint Boulevard, New Orleans, LA 70124 USA

**Keywords:** Colon cancer cell, Cytotoxicity, Gene expression, Lipopolysaccharide

## Abstract

**Objective:**

Lipopolysaccharide (LPS) is a major cell wall component of gram-negative bacteria. Colon bacteria contribute to LPS which promotes colon cancer metastasis. The objective of this study was to survey the effect of LPS on cell viability and gene expression of 55 molecular targets in human colon cancer cells.

**Results:**

LPS did not affect the viability of COLO 225 cells under the culture conditions but affected the expression of a number of genes important in inflammatory responses and cancer development. LPS increased TTP family, GLUT family and DGAT1 mRNA levels but decreased DGAT2a and DGAT2b expression in the human colon cancer cells. LPS also increased COX2, CXCL1, ELK1, ICAM1, TNFSF10 and ZFAND5 but decreased BCL2L1, CYP19A1 and E2F1 mRNA levels in the colon cancer cells. These data suggest that LPS has profound effects on gene expression in human colon cancer cells.

## Introduction

Colon cancer is one of the deadliest diseases in the World. The risk of developing colorectal cancer is approximately 4.0% for men and women in 2021 during the lifetime (https://www.cancer.org/cancer/types/colon-rectal-cancer/about/key-statistics.html). It is urgently needed to fully understand the mechanism of developing colon cancer and explore ways to ease the burden of the healthcare crisis.

Lipopolysaccharide (LPS) is a major cell wall component of gram-negative bacteria. Intact LPS is made up of three structural components [1]: a hydrophobic lipid section; a hydrophilic core polysaccharide chain, and a repeating hydrophilic O-antigenic oligosaccharide side chain. LPS is a heat-stable endotoxin which normally protects gram-negative bacteria against bile salts and lipophilic antibiotics.

LPS was proposed to have antitumor effect in several experimental models [2]. A number of studies explored the effect of LPS on gene expression in colon cancer cells, but they were focused on a few targets in the reported research. LPS induced TGFβ and HGF production [3], promoted NFkB (NFkappaB) activation [4] and increased the migratory capacity [5] in colon cancer cells.

The objective of this study was to survey the effect of LPS on cell viability and gene expression of 55 molecular targets in human colon cancer cells. The 55 molecular targets belong to several important pathways, whose expression is affected by the plant toxin gossypol in cancer cells [6–14] and macrophages [15, 16] or regulated by ZFP36/TTP in tumor cells [17–25] and macrophages [26, 27], as well as cinnamon polyphenol extract [28, 29] (Table 1). The results showed that LPS had minimal effect on cell viability but had a profound effect on gene expression at the mRNA levels in the human colon cancer cells.

**Table 1:** Basal mRNA level, reference mRNA and LPS effects on gene expression

ID	mRNA	Name	DMSO (n = 18)		LPS (n = 24)		LPS/DMSO
			Mean ± Std	Fold	Mean ± Std	Fold	Fold
H1	Ahrr1	Aryl hydrocarbon receptor	33.91 ± 1.22	*0.05*	37.63 ± 3.98	*0.00*	0.06
H2	Bcl2	B-cell lymphoma 2	**29.68 ± 1.02**	**1.00**	**29.02 ± 1.16**	**1.00**	1.00
H3	Bcl2l1	B-cell lymphoma 2 like 1	28.10 ± 2.27	***2.99***	27.72 ± 1.67	***2.45***	0.86
H4	Bnip3	BCL2 protein-interacting protein 3	27.94 ± 1.04	***3.32***	26.78 ± 1.50	***4.72***	1.30
H5	Cd36	Cluster of differentiation 36/fatty acid translocase	28.74 ± 1.25	1.92	27.65 ± 1.25	***2.58***	1.33
H6	Claudin1	Maintain tissue integrity and water retention	30.65 ± 5.90	0.51	31.23 ± 5.28	*0.22*	0.33
H7	Cox1	Cyclooxygenase 1	37.05 ± 5.88	*0.01*	39.19 ± 3.49	*0.00*	0.03
H8	Cox2	Cyclooxygenase 2	30.28 ± 1.33	0.66	31.79 ± 3.07	*0.15*	0.30
H9	Csnk2a1	Casein kinase 2 alpha 1	26.35 ± 2.10	***10.05***	26.12 ± 1.56	***7.43***	0.71
H10	Ctsb	Cathepsin B	28.47 ± 3.00	***2.31***	28.97 ± 2.31	1.03	0.53
H11	Cxcl1	Chemokine (C-X-C motif) ligand 1	32.81 ± 2.65	*0.11*	35.27 ± 5.69	*0.01*	0.18
H12	Cyclind1	Cyclin D1	34.88 ± 6.68	*0.03*	33.91 ± 4.96	*0.03*	**2.59**
H13	Cyp19a1	Cytochrome P450 family 19 subfamily A member 1	31.96 ± 3.39	*0.21*	28.56 ± 3.56	1.37	**26.40**
H14	Dgat1	Diacylglycerol O-acyltransferase 1	29.59 ± 1.93	1.06	30.17 ± 2.53	*0.45*	0.46
H15	Dgat2a	Diacylglycerol O-acyltransferase 2a	32.30 ± 2.13	*0.16*	33.30 ± 4.47	*0.05*	0.25
H16	Dgat2b	Diacylglycerol O-acyltransferase 2b	31.43 ± 1.71	*0.30*	31.19 ± 2.49	*0.22*	0.66
H17	E2f1	E2F transcription factor 1	29.82 ± 1.01	0.91	30.05 ± 2.75	*0.49*	0.57
H18	Elk1	ETS transcription factor	30.85 ± 2.79	*0.44*	31.73 ± 2.09	*0.15*	0.55
H19	Fas	Fas cell surface death receptor	31.60 ± 5.28	*0.26*	33.18 ± 4.49	*0.06*	0.26
H20	Gapdh	Glyceraldehyde-3-phosphate dehydrogenase	24.83 ± 4.17	***28.71***	25.07 ± 3.15	***15.46***	0.48
H21	Glut1	Glucose transporter 1	27.48 ± 2.72	***4.57***	29.21 ± 3.80	0.87	0.22
H22	Glut2	Glucose transporter 2	29.45 ± 2.00	1.17	29.05 ± 3.75	0.97	0.65
H23	Glut3	Glucose transporter 3	28.38 ± 1.21	***2.45***	27.78 ± 1.63	***2.35***	1.31
H24	Glut4	Glucose transporter 4	40.16 ± 5.08	*0.00*	41.70 ± 6.15	*0.00*	0.31
H25	Hif1a	Hypoxia inducible factor 1 subunit alpha	27.78 ± 2.29	***3.72***	27.67 ± 2.50	***2.53***	0.96
H26	Hmgr	3-Hydroxy-3-methylglutaryl-CoA reductase	27.85 ± 1.94	***3.54***	27.33 ± 1.25	***3.22***	0.94
H27	Hmox1	Heme oxygenase 1	30.11 ± 1.35	0.74	29.39 ± 1.23	0.77	0.96
H28	Hua	Human antigen a	32.98 ± 3.77	*0.10*	32.39 ± 3.71	*0.10*	0.75
H29	Icam1	Intercellular adhesion molecule 1/CD54	34.27 ± 4.65	*0.04*	36.49 ± 5.75	*0.01*	0.09
H30	Inos	Inducible nitric oxide synthase	ud	ud	ud	ud	ud
H31	Insr	Insulin receptor	29.99 ± 3.43	0.81	31.64 ± 3.23	*0.16*	0.29
H32	Il2	Interleukin 2	31.69 ± 1.08	*0.25*	30.31 ± 1.23	*0.41*	1.43
H33	IL6	Interleukin 6	29.51 ± 1.21	1.12	27.60 ± 1.22	***2.67***	1.95
H34	IL8	Interleukin 8	29.37 ± 1.08	1.24	28.92 ± 1.79	1.07	0.99
H35	Il10	Interleukin 10	36.16 ± 9.42	*0.01*	34.04 ± 11.21	*0.03*	1.10
H36	Il12	Interleukin 12	38.14 ± 3.63	*0.00*	32.53 ± 7.75	*0.09*	**20.16**
H37	Il16	Interleukin 16	28.45 ± 1.13	***2.33***	27.04 ± 4.88	***3.94***	1.73
H38	Il17	Interleukin 17	29.90 ± 1.30	0.85	28.92 ± 1.77	1.07	1.02
H39	Leptin	Body fat and obesity hormone	30.46 ± 5.47	0.58	28.98 ± 1.34	1.03	1.29
H40	Map1lc3a	Microtubule-associated proteins 1 light chain 3A	30.03 ± 1.82	0.78	29.03 ± 1.56	0.99	1.08
H41	Map1lc3b	Microtubule-associated proteins 1 light chain 3B	26.60 ± 1.64	***8.44***	26.93 ± 2.65	***4.25***	0.51
H42	Nfkb	Nuclear factor kappa B	31.25 ± 3.21	*0.34*	32.91 ± 5.03	*0.07*	0.28
H43	P53	Tumor suppressor	31.18 ± 2.46	*0.35*	30.71 ± 1.61	*0.13*	0.92
H44	Pim1	Proto-oncogene serine/threonine-protein kinase	29.42 ± 0.99	1.19	29.49 ± 1.57	0.72	0.59
H45	Pparr	Peroxisome proliferator-activated receptor gamma	29.36 ± 1.54	1.24	29.89 ± 1.36	0.54	0.62
H46	Rab24	Ras-related oncogene 24	41.98 ± 2.85	*0.00*	44.31 ± 5.63	*0.00*	0.15
H47	Rpl32	Ribosomal protein L32 (60S ribosomal unit)	24.59 ± 3.89	***33.88***	24.98 ± 3.10	***16.40***	0.55
H48	Tnf	Tumor necrosis factor	31.25 ± 1.76	*0.34*	30.44 ± 1.55	*0.37*	1.03
H49	Tnfsf10	Tumor necrosis factor superfamily, member 10	28.24 ± 1.68	***2.71***	28.06 ± 1.41	1.94	0.83
H50	Ulk2	Unc-51 like autophagy activating kinase 2	29.54 ± 1.02	1.10	28.33 ± 1.36	1.61	1.27
H51	Vegf	Vascular endothelial growth factor	37.19 ± 6.99	*0.01*	37.26 ± 7.71	*0.00*	0.82
H52	Zfand5	Zinc finger AN1-type containing 5	27.47 ± 1.41	***4.61***	26.95 ± 1.50	***4.20***	1.00
H53	Zfp36	Zinc finger protein 36	29.04 ± 2.01	1.55	29.15 ± 1.85	0.91	0.69
H54	Zfp36l1	Zinc finger protein 36 like 1	29.78 ± 3.02	0.93	29.67 ± 2.70	*0.64*	0.80
H55	Zfp36l2	Zinc finger protein 36 like 2	41.81 ± 3.74	*0.00*	42.67 ± 2.47	*0.00*	0.37

The fold was calculated using the mean data. Bold with italics: Genes with mRNA levels at least twofold of Bcl2. Italics: Genes with mRNA levels less than 50% of Bcl2

*ud* undetected

## Main text

### Methods

Human colon cancer cells (COLO 205) were maintained in RPMI-1640 medium (Gibco) containing 10% (v:v) fetal bovine serum, 0.1 million units/L penicillin, 100 mg/L streptomycin, and 2 mmol/L l-glutamine at 37 °C with 5% CO_2_. Cancer cells (0.5 mL) were subcultured at 1 × 10^5^ cells/mL density and treated for 2 and 24 h with 0–1000 ng/mL of LPS extracted from *E. coli* serotype K235 and purified by gel filtration (Sigma, St. Louis. MO) (“0” treatment represents the control with 1% DMSO in all treatments). Cell cytotoxicity was determined by spectrophotometer at A570 nm using the MTT based-In Vitro Toxicology Assay Kit (Sigma) [30].

The effect of LPS on gene expression was evaluated by quantitative real-time PCR analysis (qPCR). Fifty-five genes were selected for qPCR analysis (Table 1). Human colon cancer cells in triplicate were treated with LPS for 8 h. RNA isolation and cDNA synthesis were performed as described [31]. The SYBR Green qPCR assays were described previously [32, 33]. BCL2 mRNA was selected as the internal reference based on our previously analysis [14] and date presented in the “Results” section. The 2^−Δ*CT*^ and 2^−ΔΔ*CT*^ method of relative quantification was used to determine the fold change in expression [34]. The data represent the mean and standard deviation (the number of independent qPCR data ‘n’ is indicted in the tables and figure legends).

## Results

### Effect of LPS on cell viability

MTT method assessed cell cytotoxicity of human colon cancer cells (COLO 225) after the cells were treated with up to 1000 ng/mL of LPS for 2 and 24 h. MTT assay did not show significant effect of LPS on the viability of the human colon cancer cells under the culture conditions (Data not shown).

### Basal expression level

To provide a basis for gene expression comparison in the colon cancer cells, the relative mRNA levels of 55 genes were estimated by SYBR Green qPCR assay. The qPCR assay showed that the cycle of threshold (C_T_) of BCL2 mRNA was 29.68 ± 1.02 (mean ± standard deviation, n = 18, means the calculation was performed from 18 independent qPCR data) (Table 1). GAPDH and RPL32 mRNA levels were the most abundant with 29- and 34-fold of BCL2 mRNA, respectively. INOS mRNA was undetectable. AHRR1, COX1, CYCLIND1, GLUT4, ICAM1, IL10, IL12, RAB24, VEGF and ZFP36L2 mRNA levels were detected with less than 5% of BCL2 mRNA in the colon cancer cells (Table 1).

### Selection of reference mRNA

The ideal reference gene for qPCR assay is stably expressed under the experimental conditions. This can be estimated by the standard deviations among the treatments. The less of standard deviation of C_T_ among the LPS treatments indicates the more stable expression of the gene. The C_T_ value of BCL2 mRNA was 29.02 ± 1.16 (mean ± standard deviation, n = 24), one of the least varied mRNAs (Table 1). GAPDH and RPL32 mRNAs are widely used as references for qPCR assays in other mammalian cells such as adipocytes and macrophages [28, 29, 35, 36], but GAPDH and RPL32 mRNA levels had much larger standard deviations (Δ*CT* was 3.15 and 3.10, respectively) and the most abundantly expressed with 15.5- and 16.4-fold of BCL2 mRNA, respectively in the human colon cancer cells (Table 1). The large standard deviations and high expression levels of GAPDH and RPL32 mRNAs made them not good internal references for qPCR assays in the human colon cancer cells. BCL2 was among the least regulated genes by LPS and therefore suitable as the internal reference for the qPCR analyses.

### Effect of LPS on overall gene expression

To provide a general idea how these genes were expressed in the cultured colon cancer cells with or without LPS treatment, the pooled qPCR data were analyzed using BCL2 mRNA as the internal reference and DMSO treatment as the sample control. LPS upregulated the expression of three mRNAs with at least twofold of the control but decreased the expression of 16 mRNAs with less than 50% of the control. The up-regulated 3 mRNAs were CYCLIND1, CYP19A1 and IL12 (Table 1). The down-regulated 17 mRNAs were AHRR1, CLAUDIN1, COX1, COX2, CXCL1, DGAT1, DGAT2a, FAS, GAPDH, GLUT1, GLUT4, ICAM1, INSR, NFKB, RAB24 and ZFP36L2 (Table 1). However, it is worth mentioning that the expression patterns based on pooled data from various concentrations may not completely in agreement with those of the detailed analysis of pair-wised comparison between the treatment and DMSO control as detailed below.

### Effect of LPS on gossypol-reported gene expression

**Fig. 1:**
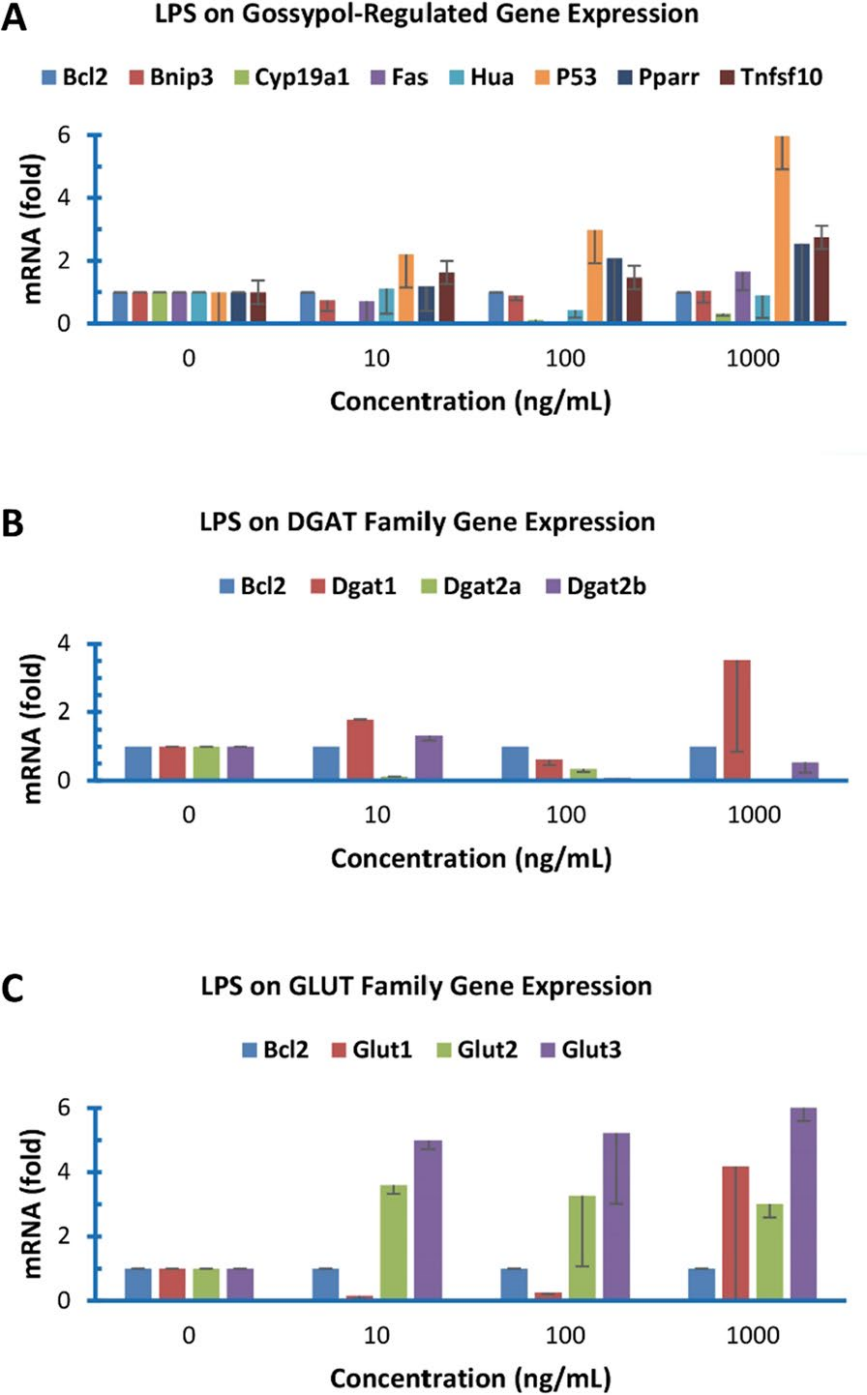
Effect of LPS on the expression of gossypol-regulated genes as well as DGAT and GLUT family genes

Several genes were shown previously to be regulated by plant toxin gossypol in cancer cells and macrophages. Here, we analyzed the expression of the same group of genes including BNIP3, CYP19A1, FAS, HuA, P53, PPARR and TNFSF10 genes under various concentrations of LPS in the colon cancer cell line using BCL2 as the internal reference gene [31]. In general, this group of genes were expressed lower than BCL2 except BINP3 and TNFSF10 (Table 1). LPS increased mRNA levels of P53, PPARR and TNFSF10 genes but decreased that of CYP19A1 gene (Fig. 1A). The effects of bacterial endotoxin LPS on the expression of this group of genes were different from those of the plant toxin gossypol which inhibited the expression of all these genes except PPARR gene to a large extent in the same human colon cancer cells [31].

### Effect of LPS on DGAT gene expression

Diacylglycerol acyltransferases (DGATs) catalyze the rate-limiting step of triacylglycerol biosynthesis by esterifying *sn*-1,2-diacylglycerol with a long-chain fatty acyl-CoA. DGAT2 mRNA is the major DGAT mRNA in mouse adipocytes and macrophages [33, 37], but DGAT1 mRNA was the major form in the human colon cancer cells (Table 1). LPS treatment under higher concentration increased DGAT1 mRNA levels but decreased DGAT2a and DGAT2b expression in the human colon cancer cells (Fig. 1B).

### Effect of LPS on GLUT gene expression

Glucose transporter (GLUT) family proteins are responsible for glucose uptake in mammalian cells. GLUT1 mRNA was the major form of GLUT mRNAs but GLUT4 mRNA was barely detected in the colon cancer cells (Table 1). LPS treatment significantly increased GLUT2 and GLUT3 mRNA levels but only high concentration of LPS at 1000 ng/mL increased GLUT1 mRNA level (Fig. 1C).

### Effect of LPS on TTP and TTP-mediated gene expression

**Fig. 2:**
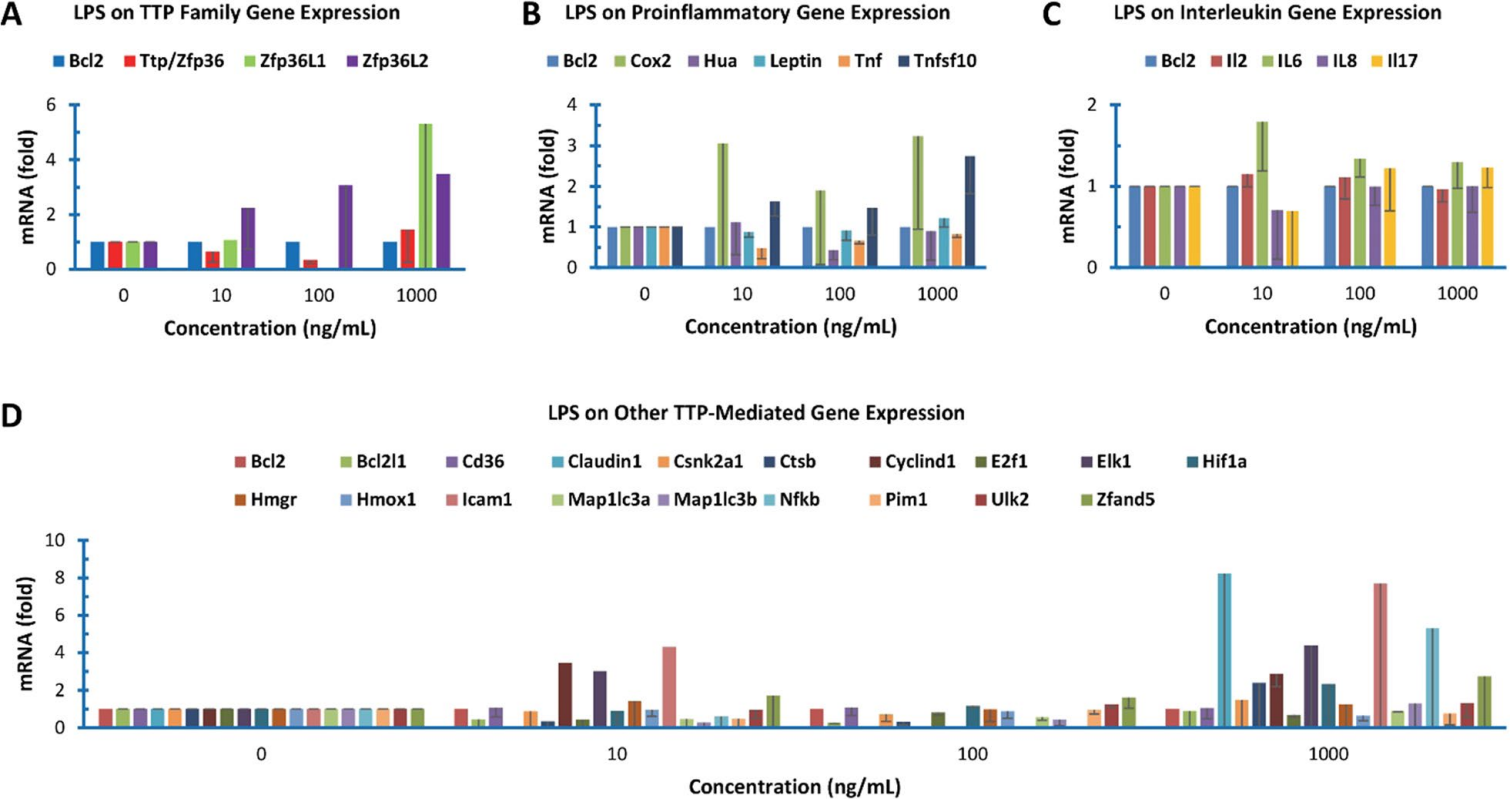
Effect of LPS on TTP and IL, proinflammatory and other family gene expression

Tristetraprolin (TTP/ZFP36) family proteins control the mRNA stability of some cytokines [38]. qPCR showed that TTP and ZFP36L1 genes were expressed in similar levels but ZFP36L2 mRNA was barely detectable in the colon cancer cells (Table 1). ZFP36, ZFP36L1 and ZFP36L2 mRNA levels were generally increased in the colon cancer cells by high concentration of LPS treatment (Fig. 2A).

### Effect of LPS on proinflammatory gene expression

TTP family proteins down-regulate the stability of several proinflammatory cytokine and enzyme mRNAs including those coding for tumor necrosis factor-alpha (TNFα) [39], granulocyte–macrophage colony-stimulating factor/colony-stimulating factor 2 (GM-CSF/CSF2) [40] and cyclooxygenase 2/prostaglandin-endoperoxide synthase 2 (COX2/PTGS2) [24]. qPCR showed that all the tested proinflammatory mRNAs except TNFSF10 mRNA were expressed much lower than that of TTP in the colon cancer cells (Table 1). LPS increased COX2 and TNFSF10 mRNA levels but did not exhibit significant effect on HuA, LEPTIN and TNF mRNA levels in the human colon cancer cells (Fig. 2B). COX1 and VEGF mRNA levels were too low to be reliable (Table 1).

### Effect of LPS on IL gene expression

TTP family proteins also regulate the stability of several interleukin (IL) mRNAs coding for IL2 [41], IL6 [42], IL8 [43], IL10 [44], IL12 [45], IL16 [23] and IL17 [46]. SYBR Green qPCR showed that IL10 and IL12 mRNAs were barely expressed and IL2 mRNA was low, whereas the other ILs were expressed in similar levels to TTP, which were several fold higher than IL2 mRNA in the human colon cancer cells (Table 1). The qPCR assays showed that LPS did not have significant effect on IL mRNA levels in the colon cancer cells (Fig. 2C).

### Effect of LPS on TTP-targeted other gene expression

A number of other TTP-mediated mRNAs have been reported in the literature. The basal levels of some mRNAs were higher than that of TTP mRNA (BCL2L1, CSNK2A1, HIF1a and ZFAND5) but the others were lower than that of TTP mRNA (AHRR1, CXCL1, E2F1, ELK1, HMOX1 and ICAM1) (Table 1). qPCR showed that LPS increased CXCL1, ELK1, ICAM1 and ZFAND5 mRNA levels, but decreased BCL2L1 and E2F1 mRNA levels in the colon cancer cells (Fig. 2D).

## Discussion

Colon bacteria contribute to a large quantity of LPS which could promote colon cancer metastasis. In this study, we surveyed the effect of LPS on cell viability and expression of 55 genes at the mRNA levels in human colon cancer cells. The data confirmed that BCL2 was the most stable mRNA among the 55 mRNAs and suitable as the reference mRNA for qPCR analyses in human colon cancer cells [31]. We observed that LPS did not affect the viability of the cells but affected the expression of a number of genes important in inflammatory responses and cancer development under the culture conditions.

The following findings are worthy of discussion. (1) High concentration of LPS increased TTP family gene expression in the human colon cancer cells, in agreement with the previous results using mouse macrophages [29, 47]. (2) LPS increased GLUT1, GLUT2 and GLUT3 mRNA levels in the human colon cancer cells, suggesting that LPS maybe able to increase glucose transport into the cancer cells since GLUT family proteins are responsible for glucose uptake in mammalian cells [27, 33]. (3) LPS treatment under higher concentration increased DGAT1 mRNA levels (the major form of DGATs) but decreased DGAT2a and DGAT2b expression in the human colon cancer cells, suggesting that LPS has limited effect on triacylglycerol biosynthesis in the colon cancer cells. (4) LPS increased COX2 mRNA levels in this study, in contrast to a previous study [48], which might be due to the cell type (COLO 225 vs. Coco-2) and/or detection methods (qPCR vs. western blot) used in the two studies. 5) LPS did not show any significant effect on HIF1a gene expression in COLO 225 cells, similar to those using MC-38 mouse colon cancer cells [49]. 6) LPS did not have significant effect on IL gene expression in this study, similar to those showing that LPS does not increase IL6, IL8 and IL15 expression in two human colon cancer cell lines [3], but differ from two reports about LPS effect on IL6 and IL8 mRNA levels in HT-29 cells [50, 51].

## Limitations

A few limitations of this study are worthy of mentioning. First, the data were generated from one colon cancer cell line (COLO 225). It could be valuable to expand the research with other cancer cell lines. Second, the dosage effect of LPS on mRNA levels was not strong and the standard deviations were large in some assays probably due to extremely sensitive qPCR assays. Third, it could be great to confirm mRNA data at the protein level. Finally, there is no functional analysis of LPS on intermediate steps between mRNA changes and cell viability. It is author’s aim to present initial observations rather than in-depth study in this manuscript. Hopefully, more detailed studies could be performed when more resources are available for this type of study.

## Data Availability

The datasets generated during the current study are available in the NIH Gene Expression Omnibus (GEO) Database, accession number GSE200980 (https://www.ncbi.nlm.nih.gov/geo/query/acc.cgi?acc=GSE200980. Materials are available from the author upon request.
